# Abnormal pairing of X and Y sex chromosomes during meiosis I in interspecific hybrids of *Phodopus campbelli* and *P. sungorus*

**DOI:** 10.1038/srep09435

**Published:** 2015-03-24

**Authors:** Satoshi Ishishita, Kazuma Tsuboi, Namiko Ohishi, Kimiyuki Tsuchiya, Yoichi Matsuda

**Affiliations:** 1 Laboratory of Animal Genetics, Department of Applied Molecular Biosciences, Graduate School of Bioagricultural Sciences, Nagoya University, Furo-cho, Chikusa-ku, Nagoya, Aichi 464-8601, Japan; 2 Laboratory of Animal Cytogenetics, Graduate School of Science, Hokkaido University, North 10 West 8, Kita-ku, Sapporo, Hokkaido 060-0810, Japan; 3 Applied Biology Co. Ltd., Minato-ku, Tokyo 107-0062, Japan

## Abstract

Hybrid sterility plays an important role in the maintenance of species identity and promotion of speciation. Male interspecific hybrids from crosses between Campbell's dwarf hamster (*Phodopus campbelli*) and the Djungarian hamster (*P. sungorus*) exhibit sterility with abnormal spermatogenesis. However, the meiotic phenotype of these hybrids has not been well described. In the present work, we observed the accumulation of spermatocytes and apoptosis of spermatocyte-like cells in the testes of hybrids between *P. campbelli* females and *P. sungorus* males. In hybrid spermatocytes, a high frequency of asynapsis of X and Y chromosomes during the pachytene-like stage and dissociation of these chromosomes during metaphase I (MI) was observed. No autosomal univalency was observed during pachytene-like and MI stages in the hybrids; however, a low frequency of synapsis between autosomes and X or Y chromosomes, interlocking and partial synapsis between autosomal pairs, and γ-H2AFX staining in autosomal chromatin was observed during the pachytene-like stage. Degenerated MI-like nuclei were frequently observed in the hybrids. Most of the spermatozoa in hybrid epididymides exhibited head malformation. These results indicate that the pairing of X and Y chromosomes is more adversely affected than that of autosomes in *Phodopus* hybrids.

Intergeneric and interspecific hybrids in vertebrates generally exhibit sterility and/or developmental dysgenesis, which is considered to prevent successful interbreeding between different species or between highly genetically differentiated populations, maintain the identity and uniqueness of species, and promote speciation^1^. Heterogametic sex-preferred sterility or lethality in interspecific hybrids has been known as Haldane's rule: “When in the F_1_ offspring of two different animal races one sex is absent, rare, or sterile, that sex is the heterozygous (heterogametic) sex.”^2^ Such sexual dimorphism of reproductive failure has frequently been reported in interspecific crosses in the Mammalia and Aves, in which postzygotic breakdown occurs more seriously in the heterogametic sex^3^. In mammals, interspecific hybrids have been obtained in many genera, such as *Thrichomys* and *Graomys* of the Rodentia, *Petrogale* of the Marsupialia, and *Equus* of the Perissodactyla; the sterility of these hybrids has been examined histologically and cytogenetically in testes, which generally exhibit spermatogenic disruptions such as meiotic arrest in spermatocytes, decreased sperm number, and sperm malformation^4^,^5^,^6^,^7^. In birds, sterility or lethality is more noticeable in interspecific or intergeneric F_1_ hybrid females^3^.

The Campbell's dwarf hamster (*Phodopus campbelli*) and the Djungarian hamster (*P. sungorus*) (Cricetidae, Rodentia) are classified as different species by morphological and molecular phylogenetic analyses, and their divergence time is estimated to be 0.8–1.0 million years^8^,^9^,^10^. These species have identical diploid chromosome numbers [2n = 28, fundamental number of chromosomal arms (NF) = 51]^11^,^12^, and the size and morphology of the chromosomes are similar; structural differences are only found for the locations and sizes of C-heterochromatin blocks on some chromosomes^13^. *P. sungorus* inhabits Kazakhstan, northwestern China, and the outskirts of these areas, while *P. campbelli* lives in steppe and semidesert areas from central to northern Asia, including Mongolia, Tuva, northeast China, and the Altai mountains. The habitats of these species are divided by the Altai-Sayan Mountains^10^; therefore, the hamsters do not interbreed in nature. However, these closely related species do crossbreed in captivity and produce vital progeny: the male offspring of these interspecific crosses are sterile, but female offspring are fertile^14^,^15^. Previous studies have shown abnormal meiotic and postmeiotic phenotypes, such as decreased testes weight, fewer and malformed spermatozoa, more frequent chromosome pairing errors at prophase, X-Y dissociation at MI, and the degeneration of metaphase I (MI) spermatocytes, in F_1_ hybrids between *P. sungorus* females and *P. campbelli* males, as well as in reciprocal F_1_ hybrids^14^,^15^. However, molecular cytogenetic examination of chromosome pairing and meiotic double-stranded DNA break (DSB) repair has not yet been performed in these offspring, and details regarding spermatozoa morphology have not yet been reported.

To further understand which processes of gametogenesis are impaired in hybrid *Phodopus* males, we performed histological analysis of testes, histochemical detection of apoptosis in seminiferous tubules, light microscopy of Giemsa-stained spermatocyte nuclei, and immunofluorescence analysis of meiotic chromosomes with antibodies to SYCP1 and SYCP3, proteins of the synaptonemal complex (SC) that mediate the attachment of homologous chromosomes^16^. We performed immunofluorescence analysis of meiotic chromosomes with an antibody to the phosphorylated histone H2AFX (γ-H2AFX), a marker for DSBs, unsynapsed chromosomal regions, and the XY body, which is a sex chromosome-specific meiotic chromatin domain^17^,^18^,^19^,^20^. We also conducted light microscopy of spermatozoa in F_1_ hybrids between *P. campbelli* females and *P. sungorus* males. We discuss the molecular and cellular mechanisms underlying abnormal gametogenesis in *Phodopus* hybrid males.

## Results

### Small testes and abnormal seminiferous epithelia in the hybrids

**Figure 1:**
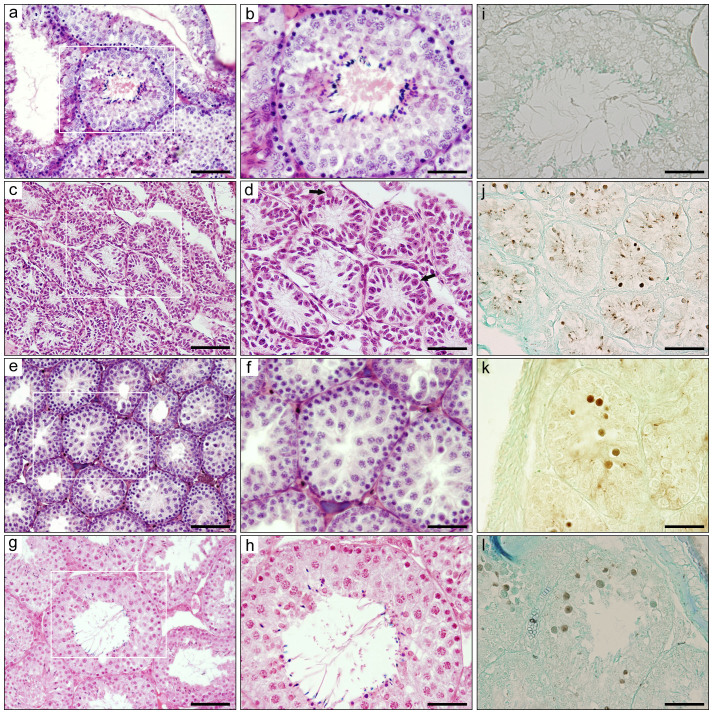
Histological and histochemical analysis of testes. (a–h) Hematoxylin and eosin-stained testicular cross-sections of *Phodopus sungorus* (a, b) and Type A (c, d), Type B (e, f), and Type C (g, h) hybrids. b, d, f, h are high-magnification images of the square areas in a, c, e, and g, respectively. In Type A hybrid testes, seminiferous tubules were thin, and a small number of cells were contained in these tubules (c, d). Arrows in d indicate primary spermatocytes. In Type B hybrid testes, primary spermatocytes were accumulated and few or no secondary spermatocytes and postmeiotic cells were present (f). Type C hybrid testes contained a small number of spermatozoa (h). i–l Detection of apoptotic cells in seminiferous tubules in *P. sungorus* (i) and Type A (j), Type B (k), and Type C (l) hybrid testes using TdT-mediated dUTP nick-end labeling (TUNEL). The apoptotic spermatocytes were darkly stained. Many apoptotic spermatocytes were observed in the hybrid testes, whereas few or no apoptotic cells were observed in *P. sungorus*. The tissue sections were counterstained with methyl-green. Scale bars represent 100 μm (a, c, e, g) and 50 μm (b, d, f, h–l).

F_1_ hybrid males were obtained from crosses between *P. campbelli* females and *P. sungorus* males in the present study, as the reverse mating leads to serious dystocia that results in embryonic death before parturition, caused by the overgrowth of hybrid embryos and placental hypertrophy^21^ (data not shown). The testis weight was lower in the hybrids (176.8 ± 143.5 mg) (mean ± s.d.) than in *P. campbelli* (1109.3 ± 145.3 mg) (Welch's *t*-test, *P* < 0.01) and *P. sungorus* (957.0 ± 67.5 mg) (*P* < 0.0001), and the relative testis weight [testis weight (g)/body weight (g) × 100] was also lower in the hybrids (0.89 ± 0.72) than in *P. campbelli* (3.33 ± 0.38) (*P* < 0.01) and *P. sungorus* (2.95 ± 0.43) (*P* < 0.01) (Table S1). Large individual differences were found in both the testis weight and relative testis weight in the hybrids (Fig. S1). Some hybrid males (Type A) exhibited very small testes (27.2 ± 8.7 mg) (Table S1) in which seminiferous tubules were thin and fewer cells were present in the seminiferous epithelia (Fig. 1c, d). Besides spermatogonia and Sertoli cells, a small number of primary spermatocytes were also observed in the testes. The other hybrid males could be divided into two types based on testicular phenotype. In 9 of 20 hybrid males, few or no secondary spermatocytes and postmeiotic cells were observed; however, primary spermatocytes were abundantly accumulated (Type B) (Fig. 1e, f). In the testes of Type B males, the seminiferous tubules had thin and irregular epithelia, and the arrays of germ cells were disrupted in fragile adhesion. The degenerated spermatocytes were often exfoliated into the lumen of the seminiferous tubules. The remaining 11 hybrid males accumulated primary spermatocytes at the pachytene-to-MI-like stage, as seen in the testes of Type B; however, a small number of postmeiotic cells and mature spermatozoa were also observed, leading to the classification of these males as Type C (Fig. 1g, h).

Few or no TUNEL (TdT-mediated dUTP nick-end labeling)-positive apoptotic cells were observed in the seminiferous tubules of the parental species, *P. sungorus* and *P. campbelli* (Fig. 1i). By contrast, a number of TUNEL-positive apoptotic spermatogenic cells were observed in the seminiferous tubules of hybrids with all three types of testes (Type A, Type B, and Type C) (Fig. 1j–l). The TUNEL-positive cells were primarily located in the inner area rather than the basal layer and had large and round nuclei, suggesting that these apoptotic cells were primary spermatocytes exfoliated into the lumen of the seminiferous tubules.

The testis weights of Type A (27.2 ± 8.7 mg), Type B (142.6 ± 77.9 mg), and Type C (327.2 ± 81.9 mg) hybrids were significantly lower than those of parental species (*P* < 0.01 Type A hybrids vs. *P. campbelli*; *P* < 0.01 Type A hybrids vs. *P. sungorus*; *P* < 0.01 Type B hybrids vs. *P. campbelli*; *P* < 0.001 Type B hybrids vs. *P. sungorus*; *P* < 0.01 Type C hybrids vs. *P. campbelli*; *P* < 0.001 Type C hybrids vs. *P. sungorus*) (Table S1). The relative testis weights of Type A (0.14 ± 0.05), Type B (0.72 ± 0.35), and Type C (1.65 ± 0.41) hybrids were also significantly lower than those of parental species (*P* < 0.01 Type A hybrids vs. *P. campbelli*; *P* < 0.01 Type A hybrids vs. *P. sungorus*; *P* < 0.01 Type B hybrids vs. *P. campbelli*; *P* < 0.01 Type B hybrids vs. *P. sungorus*; *P* < 0.01 Type C hybrids vs. *P. campbelli*; *P* < 0.05 Type C hybrids vs. *P. sungorus*) (Table S1). The testis weight and relative testis weight were considerably lower in Type A hybrids than in Type B and Type C hybrids, and also considerably lower in Type B hybrids than in Type C hybrids. These results suggest a correlation between testis weight and spermatogenic phenotype in hybrids, as has previously been observed^14^,^15^. Because the number of testicular cells was low in Type A hybrids, we examined meiotic situation and chromosomal configurations during prophase and MI and sperm morphologies using Type B and Type C hybrids in the following experiments.

### X-Y dissociation and degeneration of nuclei during MI

**Figure 2:**
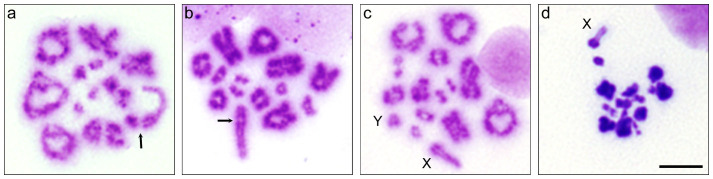
Light microscopy of metaphase spreads of primary spermatocytes. (a–d) Giemsa-stained meiotic chromosomes in primary spermatocytes of *Phodopus campbelli* (a) and F_1_ hybrids (b–d). MI spermatocytes with terminally associated X and Y chromosomes (a, b, arrows). MI spermatocyte with dissociated X and Y chromosomes (c). X and Y chromosomes are indicated by X and Y, respectively. Degenerated MI-like spermatocyte with heteropycnotic autosomes and a lightly stained univalent X chromosome (d). Scale bars represent 20 μm.

To examine the progression of meiosis I in hybrids, light microscopy of meiotic nuclei was conducted. MI-like nuclei with degenerated chromosomes were omitted from the analysis of X and Y chromosome associations. The end-to-end association of X and Y chromosomes was observed in most of the primary spermatocytes at MI in the parental species (Fig. 2a). In contrast, the X and Y chromosomes were dissociated in 93.5% and 90.2% of MI spermatocytes in Type B and Type C hybrids, respectively; these frequencies were much higher than those observed in both parental species (7.0% in *P. campbelli* and 6.0% in *P. sungorus*) (Welch's *t*-test, *P* < 0.01 Type B hybrids vs. *P. campbelli*; *P* < 0.01 Type B hybrids vs. *P. sungorus*; *P* < 1 × 10^−7^ Type C hybrids vs. *P. campbelli*; *P* < 0.001 Type C hybrids vs. *P. sungorus*) (Fig. 2c, Table 1). Autosomal dissociation was not found in either the parental species or hybrids at the MI stage. Numerous degenerated MI spermatocytes with heteropycnotic chromosomes were observed in the hybrid testes (Fig. 2d, Table 2), accounting for 63.5% and 57.4% of MI spermatocytes in Type B and Type C hybrids; these values were significantly higher than the 5.5% and 6.5% observed in *P. campbelli* and *P. sungorus* (*P* < 1 × 10^−6^ Type B hybrids vs. *P. campbelli*; *P* < 0.01 Type B hybrids vs. *P. sungorus*; *P* < 1 × 10^−9^ Type C hybrids vs. *P. campbelli*; *P* < 0.0001 Type C hybrids vs. *P. sungorus*).

### Abnormal chromosome synapsis at the pachytene-like stage

**Figure 3:**
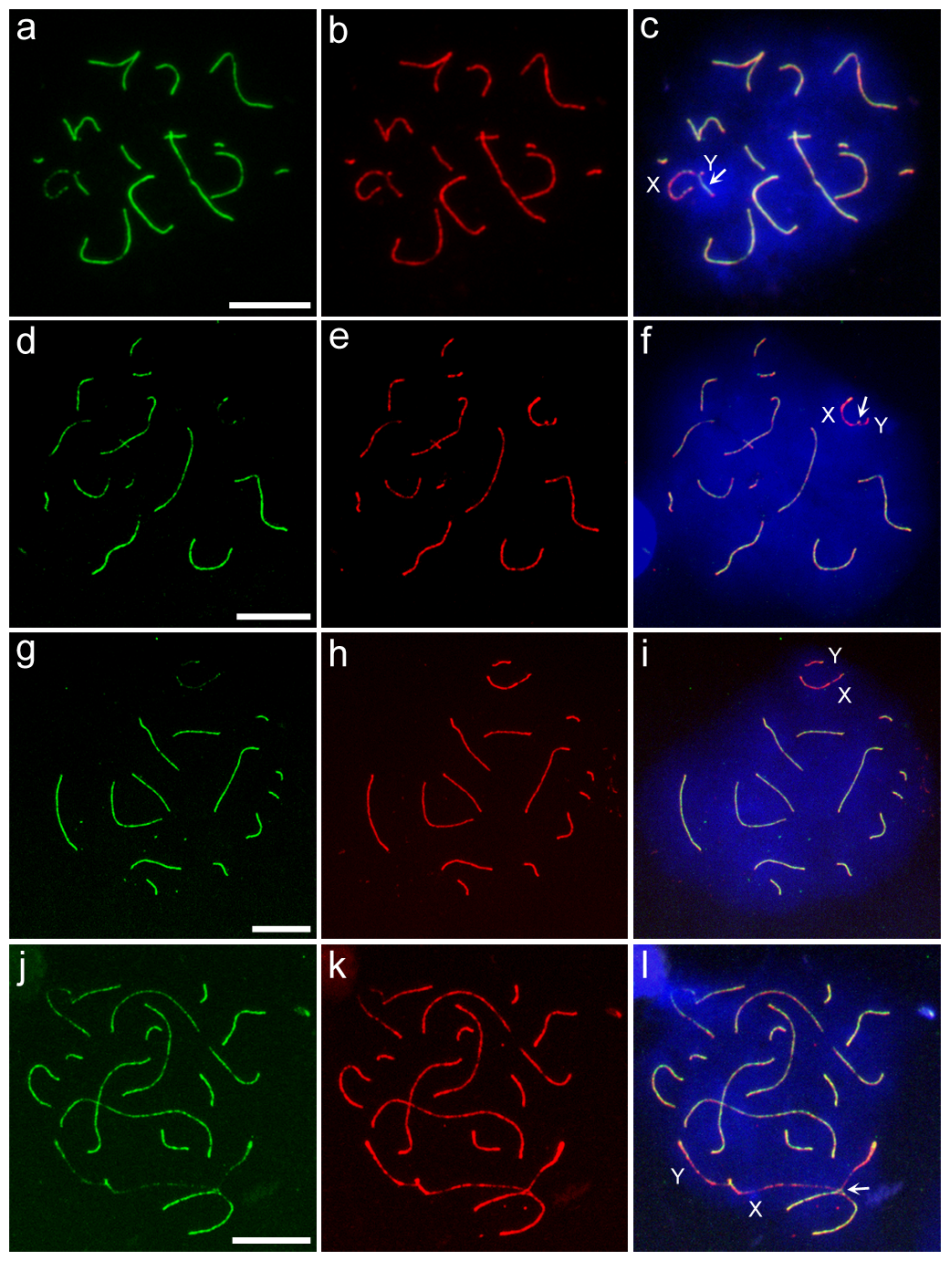
Immunocytochemical analysis of chromosome synapsis. (a–l) Immunostaining of the synaptonemal complex with SYCP1 and SYCP3 antibodies in pachytene spermatocytes of *Phodopus campbelli* (a–c) and pachytene-like spermatocytes of F_1_ hybrids (d–l). Left panels, SYCP1-stained patterns (green); middle panels, SYCP3-stained patterns (red); and right panels, merged patterns of SYCP1- and SYCP3-stained images. Spermatocytes with synaptic X and Y chromosomes (arrows) in *P. campbelli* (a–c) and hybrids (d–f). Spermatocytes with asynaptic X and Y chromosomes in the hybrid (g–i). *P.* *campbelli* X chromosome core and *P. sungorus* Y chromosome core were weakly coated with fluorescein isothiocyanate (FITC)-labeled SYCP1 antibody (g). Spermatocytes with nonhomologous pairing of X and autosomes in the hybrid (j–l). The X chromosome axis was partially synapsed with the autosomal axis (l, arrow). Scale bars represent 10 μm. Nuclei were counterstained with 4′,6-diamidino-2-phenylindole (DAPI) (blue).

To examine chromosome synapsis during the meiotic prophase, immunocytochemical analysis was performed using antibodies to SYCP1 and SYCP3, the proteins of the transverse filaments and axial/lateral elements of the synaptonemal complex, respectively. Asynapsis of X and Y chromosomes was not observed in the pachytene spermatocytes of the parental species. By contrast, asynaptic X and Y chromosomes were frequently found in the pachytene-like spermatocytes of both Type B (26.9%) and Type C (20.3%) hybrids (Fig. 3a–i, Table 3, Table S2). Notably, it was unclear whether X-Y paring in the hybrids involved homologous synapsis at pseudoautosomal regions (PARs), where reciprocal recombination between X and Y chromosomes would occur, or incomplete chromosome synapsis that was insufficient for homologous recombination, such as transient nonhomologous association at the PAR and its surrounding regions. Moreover, it was difficult to discriminate completely between the late zygotene-like and pachytene-like stages in the hybrids. The use of a marker for mid-late pachytene spermatocytes (histone H1t^22^) and a marker for the presumptive crossover sites (MLH1^16^) will be required to address these issues definitively. No autosomal univalency was observed in the hybrids; however, interlocking and partial synapsis between autosomal pairs were observed in a small fraction of the pachytene-like spermatocytes of Type B (0.8%) and Type C (0.5%) hybrids (Table 3). Both the X and Y chromosomes occasionally paired partially with asynaptic sites of the autosomal axes in the hybrids (Fig. 3j–l, Table 3). The frequencies of abnormal synapsis in autosomes, which included synapses between autosomal pairs and between autosomes and X or Y chromosomes, were much lower than those of the abnormal synapsis in X and Y chromosomes. This included asynapsis of X and Y chromosomes and synapsis between autosomes and X or Y chromosomes in both Type B (Welch's *t*-test, *P* < 0.001) and Type C (*P* < 0.05) hybrids.

### γ-H2AFX staining

**Figure 4:**
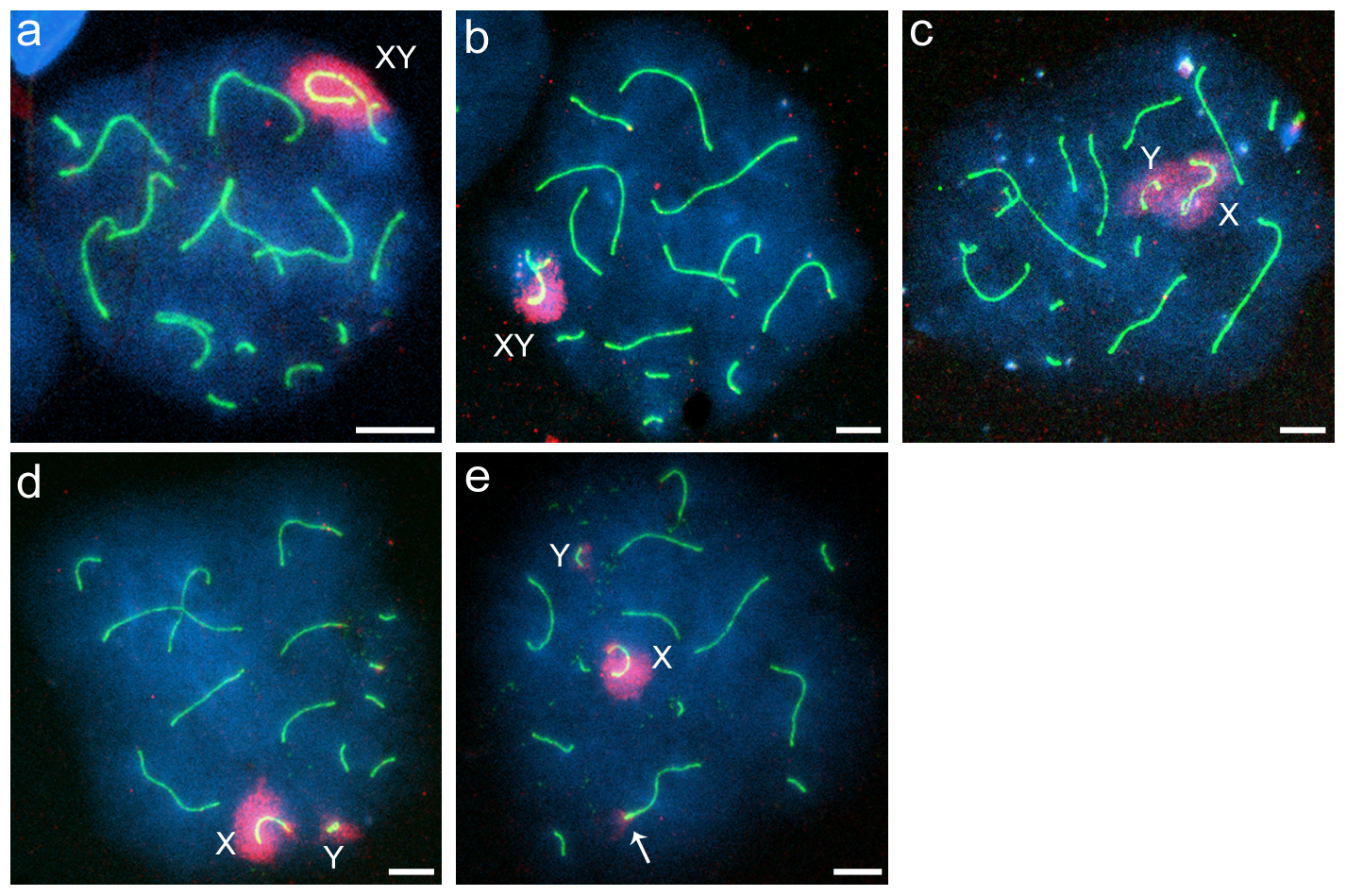
Distribution of γ-H2AFX staining in spermatocyte nuclei. (a–e) Immunocytochemical staining of γ-H2AFX and the lateral element of the synaptonemal complex in pachytene spermatocytes of *Phodopus campbelli* (a) and pachytene-like spermatocytes of F_1_ hybrids (b–e). γ-H2AFX and SYCP3 staining are indicated in red and green, respectively. Spermatocytes with synaptic X and Y chromosomes around which γ-H2AFX was normally distributed (a, b). Hybrid spermatocyte with asynaptic X and Y chromosomes (c–e). The γ-H2AFX distributions were broad (c) or separated (d, e). An autosomal pair was stained with γ-H2AFX antibody (e, arrow). Scale bars represent 10 μm.

To examine the formation of XY bodies and the presence of unrepaired DSBs and/or unsynapsed chromosomal regions in pachytene spermatocytes, immunofluorescence staining with γ-H2AFX and SYCP3 antibodies was performed. Few or no spermatocytes of the parental species showed asynapsis of the X and Y chromosomes, while this asynapsis was observed frequently in the hybrids (Table 4, Table S3). The frequencies of X-Y asynapsis in the hybrids were not significantly different from those observed by immunostaining with SYCP1 and SYCP3 antibodies (Welch's *t*-test, P > 0.05 in both Type B and Type C hybrids). γ-H2AFX staining was observed around the paired sex chromosomes, but not on autosomal chromatin, in the pachytene spermatocytes of the parental species (Fig. 4a, Table 4, Table S3). Hybrid spermatocytes with asynaptic X and Y chromosomes showed three types of XY bodies: normal, broad, and separated (Fig. 4b–d), the frequencies of which were 23.9%, 2.9%, and 3.7%, respectively, in Type B hybrids and 19.0%, 1.0%, and 0.8%, respectively, in Type C hybrids (Table 4). In contrast to the parental species, γ-H2AFX staining was also observed in the autosomal chromatin of 5.5% and 3.2% of the spermatocytes in Type B and Type C hybrids, respectively (Fig. 4e, Table 4). In most cases, autosomal γ-H2AFX staining was observed in only one or two pairs of chromosomes. The frequencies of cells exhibiting γ-H2AFX staining in autosomal chromatin were lower than those of cells exhibiting asynapsis of X and Y chromosomes in Type B and Type C hybrids. (Table 4, Table S3); however, no significant difference was detected in Type C hybrids (Welch's *t*-test, *P* < 0.05 in Type B hybrids; *P* = 0.08 in Type C hybrids). Association between the XY body and autosomes was observed in a small fraction of the pachytene-like spermatocytes of Type B (2.3%) and Type C (2.0%) hybrids (Table. S3); however, this association may be an artifact of the spreading of spermatocyte nuclei.

### Malformation of sperm heads

**Figure 5:**
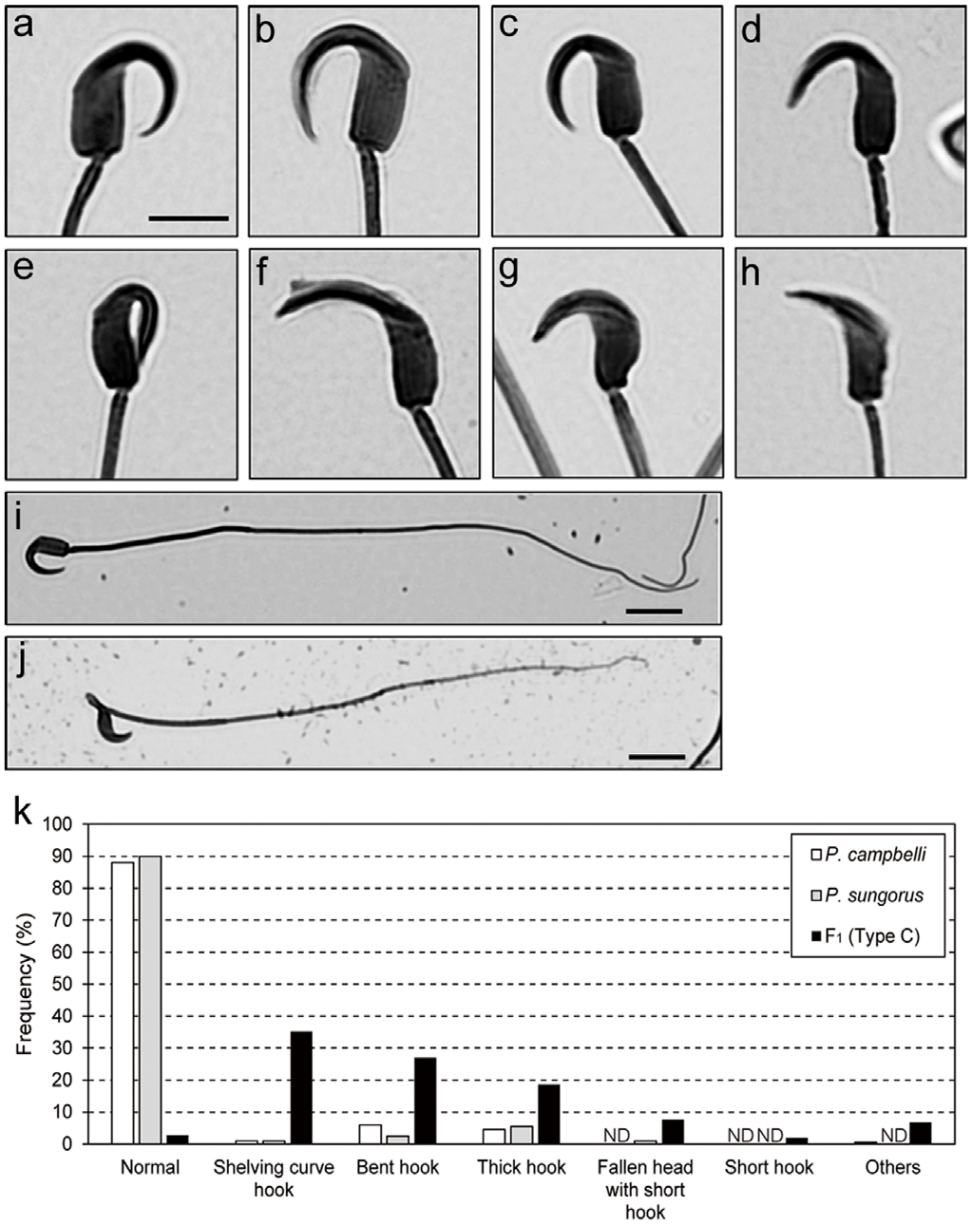
Light microscopy of epididymal spermatozoa. (a–h) Morphologies of sperm heads in parental species (a, b) and F_1_ hybrids (c–h). Normal sperm heads in *Phodopus campbelli* (a), *P. sungorus* (b), and their hybrids (c). Abnormal sperm heads in hybrids: shelving curvature of hook (d), bent hook (e), thick hook (f), fallen head with short hook (g), and short hook (h). i, j Spermatozoon with normal tail in *P. campbelli* (i) and spermatozoon with abnormal tail in the hybrid (j). Scale bars represent 5 μm (a–h) and 50 μm (i, j). k Frequencies of normal and abnormal sperm morphologies. ND, not detected.

Mature spermatozoa were collected from the caudal epididymides of parental species and Type C hybrids, and the morphological abnormalities of their sperm heads were qualified (Fig. 5, Table S4). A higher frequency of abnormal sperm heads was found in the hybrids (97.2%) compared to the parental species (12.0% in *P. campbelli* and 10.0% in *P. sungorus*). The hybrid sperm showed various morphological abnormalities: the major abnormalities were shelving curve hooks (35.2%) and bent hooks (27.0%), while the minor abnormalities included thick hooks, fallen heads with short hooks, short hooks, etc. Morphological abnormalities were also observed in the sperm tails (Fig. 5).

## Discussion

We observed high frequencies of MI nuclear degeneration and X-Y dissociation during MI in the spermatocytes of interspecific hybrids between *P. campbelli* females and *P. sungorus* males, whereas no MI spermatocytes with autosomal dissociation were observed. Moreover, a high frequency of asynaptic X and Y chromosomes was observed at the pachytene-like stage in the hybrids. In these hybrids, pachytene-like spermatocytes that exhibited abnormal autosomal synapsis were much fewer than those exhibiting abnormal synapsis in X and Y chromosomes. Considering that only one pair of sex chromosomes is present in the nucleus, compared to thirteen autosomal pairs, these results suggest the existence of a strong chromosome bias in the frequency of abnormal synapsis in hybrids. The frequency of γ-H2AFX staining in autosomes was also low, which suggests that the failure of DSB repair and/or chromosome synapsis occurs at a low frequency in autosomes. A high incidence of X-Y dissociation in F_1_ hybrids of *Phodopus* has been reported by Safronova and Vasil'eva^15^. Using electron microscopy of chromosome synapsis in these hybrids, these authors showed that pairing errors occurred at higher frequencies than in the present study for both sex chromosomes and autosomes: X-Y asynapsis was observed in 56.7% of the spermatocytes of hybrids (F_1_D) from *P. sungorus* females and *P. campbelli* males and in 37.2% of the spermatocytes of hybrids (F_1_R) from *P. campbelli* females and *P. sungorus* males. Autosomal partial asynapsis and interlocking of autosomes were observed in 19.7% of F_1_D spermatocytes and in 34.9% of F_1_R spermatocytes; association between sex chromosomes and autosomes was observed in 30.1% of F_1_D spermatocytes and in 10.9% of F_1_R spermatocytes. In our present study, γ-H2AFX was distributed around the X and Y chromosomes in the pachytene-like F_1_R spermatocytes, as well as in the spermatocytes of the parental species, even though the X and Y chromosomes were separated. This result suggests that the XY body was formed normally in terms of the phosphorylation of H2AFX in hybrids. Further investigation is necessary to clarify whether transcriptional repression of X- and Y-linked genes by meiotic sex chromosome inactivation (MSCI), which is essential for male fertility^23^, occurs normally in the hybrids. γ-H2AFX remained in the autosomal chromatin in only a small fraction of the pachytene-like spermatocytes of F_1_ hybrids, in which γ-H2AFX-positive XY bodies were formed; however, the previous study reported high frequencies of abnormal synapsis in autosomal pairs and associations between sex chromosomes and autosomes, as described above. This difference may have been caused by differences in the genetic backgrounds of the *P. campbelli* females and *P. sungorus* males used for the experiments. However, our present findings and the previous findings both found high frequencies of asynapsis for X and Y chromosomes during meiotic prophase I in the hybrids. Epididymal spermatozoa in the hybrids showed various morphological anomalies. The malformation of spermatozoa in F_1_D has also been reported previously^16^; however, to our knowledge, their morphologies have not been described. Based on our present findings, we discuss the causal factors of abnormal spermatogenesis and its molecular and cellular mechanisms in interspecific *Phodopus* hybrids.

The high frequency of asynapsis observed between X and Y chromosomes in the pachytene-like spermatocytes of the hybrids suggests that chromosome synapsis and subsequent meiotic recombination in sex chromosomes are often inhibited in hybrid spermatocytes. When spermatocytes fail to complete synapsis and recombination between homologous chromosomes, these cells are likely prevented from passing through the pachytene due to the “pachytene checkpoint” and are then eliminated by apoptosis^24^,^25^,^26^. However, previous studies of hybrid sterility in mice and mutant mice that fail in sex chromosome pairing at MI suggest that spermatocytes with asynaptic X and Y chromosomes reach the MI stage without meiotic arrest at prophase I^27^,^28^,^29^,^30^, although it remains uncertain whether all cells with asynaptic X and Y chromosomes can pass through the pachytene. Considering that X and Y chromosomes are only partially synapsed even in the pachytene spermatocytes of parental species, the pachytene checkpoint may be tolerant of the asynapsis of X and Y chromosomes in nature. The present study showed that X-Y dissociation at MI occurred at a high frequency in the *Phodopus* hybrids, as has been observed in hybrids between *Mus spretus* and laboratory mice^27^,^28^,^29^. This result suggests that spermatocytes that fail synapsis and/or recombination between X and Y chromosomes, but not between autosomes, reach MI without arrest at the pachytene. MI spermatocytes with sex chromosome univalency are thought to be eliminated efficiently by apoptosis in mice, although a small fraction of spermatocytes survive^30^. Therefore, X-Y asynapsis should decrease sperm count by preventing spermatocytes from passing through MI, which may be a primary cause of hybrid sterility. Intriguingly, X-Y dissociation at MI was more frequently observed than X-Y asynapsis at the pachytene-like stage. There are two possible explanations for this difference: first, a large number of the pachytene-like cells, in which the X and Y chromosomes underwent abnormal chromosome synapsis, such as transient nonhomologous association at the PAR and its surrounding regions, and failed to complete the subsequent meiotic recombination, may have been regarded as possessing X-Y synapsis; second, MI spermatocytes with X-Y dissociation may have been accumulated in hybrid testes due to the blockage of the cell-cycle progression at MI. Mice with a single asynaptic sex chromosome are known to exhibit accumulation of MI spermatocytes in the testes^31^. It should be noted that paring errors during prophase may represent transient events that only delay meiotic progression, and the X-Y dissociation at MI may thus result from causes other than these errors at the pachytene.

Homologous recombination repair of meiotic DSBs is essential for proper chromosome synapsis in mammals^32^. DSB-independent pairing interactions may also play roles in chromosome synapsis in mammals and other organisms^32^,^33^. Although both the roles and molecular basis of DSB-independent pairing interactions remain unclear, these mechanisms might require homology between two DNA sequences, as is required in homologous recombination^32^,^34^. In mammals, the nucleotide sequence of the PAR experiences rapid change^35^,^36^,^37^. Therefore, the sequence homology of the PAR between the X and Y chromosomes in the hybrids is likely rather low. We speculate that this decrease in sequence homology may interfere with homologous recombination and other pairing interactions, thereby causing a high frequency of X-Y asynapsis. This hypothesis requires further investigation.

Besides X-Y asynapsis, abnormal synapsis between autosomal pairs, association between sex chromosomes and autosomes, and unrepaired DSBs and/or unsynapsed chromosomal regions in autosomes was observed in the hybrids. The engulfment of completely or partially unsynapsed autosomes by XY bodies was also observed in pachytene spermatocytes of *Mus* hybrid males^38^. In spermatocytes with these abnormalities, meiotic progression is likely blocked at the pachytene by the pachytene checkpoint or transcriptional silencing of autosomal genes^18^,^19^,^40^,^41^,^42^. However, we cannot preclude the possibility that the impaired synapsis and DSB repair observed in autosomes are transient and do not prevent the cell-cycle progression beyond the pachytene.

Some hybrids (Type C) produced a small number of sperm, most of which exhibited severe abnormal morphologies; however, some of these sperm were normal. A high incidence of meiotic arrest likely decreases sperm count in the hybrids_._ This severe oligospermia and the sperm morphological abnormalities may lead to the failure of fertilization, thereby causing infertility in hybrids^42^. Further studies are required to clarify whether these malformed sperms have normal fertilization ability; however, the present findings suggest that hybrid incompatibility also affects spermiogenic processes, such as the elongation of spermatids, removal of cytoplasm from sperm heads, sperm tail formation, etc., in postmeiotic cells that have passed through the barrier at MI, as seen in *Mus* hybrids^43^,^44^. Postmeiotic transcriptional repression as a consequence of meiotic silencing of unsynapsed chromatin may be one of the main causes of abnormal spermiogenesis^39^. Alternatively, failure of postmeiotic sex chromatin repression, which is caused by disruption of MSCI at meiotic prophase I^45^, may be involved in abnormal spermiogenesis.

It should be noted that the parents used in this study were not from inbred lines, which caused individual differences in the abnormal testicular phenotypes of hybrid males. Furthermore, it should be noted that other hybrid incompatibility factors must cause spermatogenic defects in *Phodopus* hybrids beyond those that cause failures of chromosome pairing and/or recombination at meiosis I; the testicular phenotype may therefore result from the combination of abnormal phenotypes caused by these incompatibility factors. The hybrids with very small testes (Type A) are good animal models for studying the hybrid incompatibility factors that cause defects in the differentiation and/or proliferation of germ cells. The incompatibility factors that cause abnormal testicular phenotypes other than meiotic failures merit future examination.

Several explanations for Haldane's rule have been proposed. One major hypothesis is the dominance theory, which assumes that X-linked recessive alleles contribute to hybrid incompatibility^46^. In this theory, hemizygous males are more adversely affected than heterozygous females. Contrary to the dominance theory, Coyne and Orr^46^ argued that X-linked hybrid sterility genes have more adverse effects on males than females owing to the large effect of hybrid sterility genes in spermatogenesis. In mice, disruption of MSCI is a proposed cause of male-specific hybrid sterility^17^,^23^,^38^,^41^,^45^,^47^,^48^. Bhattacharyya et al.^38^ hypothesized that male sterility of intersubspecific F_1_ hybrids is caused by meiotic asynapsis due to the *cis*-acting mismatch between homologous chromosomes, which is modulated by *trans*-acting hybrid sterility genes, such as *Hst1/Prdm9* and *Hstx2*, the latter of which locates on the X chromosome. Meiotic abnormalities also occur in interspecific and intersubspecific hybrid female mice; however, the frequency of meiotic asynapsis was lower in hybrid females than in hybrid males^29^,^38^. Bhattacharyya et al.^38^ indicated that a substantial portion of pachytene oocytes exhibited pairing errors, and these oocytes were eliminated by apoptosis in hybrid females from the cross between *M. m. musculus* and *M. m. domesticus*. The authors hypothesized that the absence of MSCI and the low frequency of asynapsis in female meiosis cause the sex difference in the reproductive phenotype. The present study focused on SC formation and DSB repair during meiosis in hybrid males. Female meiosis in *Phodopus* hybrids has not yet been studied. We note the possibility that synaptic failure occurs in hybrid oocytes. The meiotic situation and meiotic chromosome configurations at prophase and MI during oogenesis should be studied in hybrid *Phodopus* females. The checkpoint systems in meiotic cells are considered to be more stringent during spermatogenesis than oogenesis, and their control mechanisms likely differ between males and females^16^,^23^,^49^,^50^,^51^. Based on these findings and the model proposed by Bhattacharyya et al.^52^, it is plausible that the less stringent MI checkpoint and absence of MSCI in oogenesis reduce the severity of the reproductive phenotype in *Phodopus* hybrid females.

In some cases, X-Y or Y-autosome incompatibility contribute to hybrid sterility^3^,^53^,^54^. A high frequency of X-Y synaptic failure was observed in previous studies of hybrid sterility in *Mus* and *Phodopus*^15^,^27^,^28^,^29^,^38^. The results of the present study strongly suggested that X-Y pairing is more adversely affected than autosomal pairing in *Phodopus* hybrids. The failure in X-Y pairing may underlies sex bias of sterility in the hybrids. Bhattacharyya et al.^38^,^52^ showed that synapsis can be recovered specifically in consubspecific but not heterosubspecific homologs in intersubspecific *Mus* hybrid males and females, which suggests that asynapsis in intersubspecific hybrids depends on sequence incompatibility between homologs. In *Phodopus* hybrids, it has been shown that backcross hybrid males obtained by crossing fertile F_1_ females with males of the parental species exhibit a reduction in synaptic failures; however, a detailed genetic analysis has not been performed^55^. Investigations of the genetic basis for XY-biased synaptic failure in *Phodopus* hybrids would provide important insight into the molecular mechanisms for hybrid male sterility and homologous chromosome pairing.

Hybrid sterility in mammals has been extensively studied using mouse models. Because Campbell's dwarf and Djungarian hamsters are small and easily bred, their interspecific hybrids would be a useful experimental model for better understanding the commonality and diversity of the genetic basis underlying reproductive isolation and Haldane's rule in rodents. Next-generation sequencing technologies, such as *de novo* and reference-based genome assembly, genotyping-by-sequencing, and restriction-site associated DNA sequencing^56^,^57^,^58^, will allow us to conduct genetic analyses of hybrid sterility in non-model organisms, such as *Phodopus* dwarf hamsters.

## Methods

### Animals

Closed colonies of *Phodopus* *sungorus* and *P. campbelli*, which were derived from breeding stocks at the Tokyo University of Agriculture, are maintained at Nagoya University. F_1_ hybrids were obtained by intercrossing between *P.* *campbelli* females and *P. sungorus* males. Males 6–8 months in age were used for the experiment. The animals were housed in plastic cages with free feeding under a 14:10-h light-dark cycle at 22°C ± 2°C. All animal care and experimental procedures were approved by the Animal Experiment Committee, Graduate School of Bioagricultural Sciences, Nagoya University (approval no. 2009043001), and the experiments were conducted according to the Regulations on Animal Experiments at Nagoya University.

### Histological analysis of testis sections

After intraperitoneal injection of a fatal dose of pentobarbital, testes were removed, fixed overnight in Bouin's solution, and then stored in 70% ethanol at room temperature until use. The fixed testes were dehydrated in a graded ethanol series, immersed sequentially in 1:1 ethanol/xylene and xylene, and then embedded in paraffin. The testes were sectioned at a thickness of 6 μm and mounted on ovalbumin-coated glass slides. The sections were deparaffinized and stained with Carazzi's hematoxylin and eosin.

### Detection of apoptosis

The apoptosis of spermatogenic cells was examined with testicular cross-sections using the TUNEL (TdT-mediated dUTP nick-end Labeling) assay^59^,^60^. Staining of apoptotic cells was performed using the ApoMark apoptosis detection kit (Exalpha Biologicals). The sections were deparaffinized following the method described above and rinsed with 1 × TBS for 5 min. Apoptotic cells were detected following the manufacturer's protocol. Slides were counterstained using methyl green after the reaction.

### Antibodies

Mouse polyclonal antibody to SYCP1 and mouse and rabbit polyclonal antibodies to SYCP3, which were provided by Dr. K. Kitada, Hokkaido University, as well as rabbit polyclonal antibody to H2AFX phosphorylated on serine residue 139 (γ-H2AFX) (Trevigen, 4411-PC-020), were used as the primary antibodies for immunostaining. For the generation of polyclonal antibody to SYCP1, the coding sequence of *SYCP1* was obtained from the total RNA of rat testis using RT-PCR. The full coding sequence of *SYCP1* was inserted into the pET102/D-TOPO vector (Life Technologies), and the recombinant protein was expressed in *Escherichia coli* and purified using Ni Sepharose 6 Fast Flow (GE Healthcare) columns under denaturing conditions. Polyclonal antibody to SYCP1 was generated from mice immunized with the recombinant protein. Methods for the production of mouse and rabbit polyclonal antibodies to SYCP3 have been described elsewhere^61^. SYCP1 and SYCP3 antibodies and γ-H2AFX antibody were used at 1:250 and 1:150 dilutions, respectively. For fluorescent staining, goat anti-mouse IgG conjugated with fluorescein isothiocyanate (FITC) (Dako, F0479) and swine anti-rabbit IgG conjugated with tetramethylrhodamine isomer R (TRITC) (Dako, R0156) were used as secondary antibodies at 1:500 and 1:1000 dilutions, respectively.

### Chromosome analysis

Meiotic chromosome preparations for the light microscopy of meiotic chromosomes were made using the air-drying method without colchicine treatment, as described previously^62^. After hypotonic treatment of the seminiferous tubules in 1% sodium citrate for 20 min at room temperature, they were placed in a fixative solution (1:1 ethanol/acetic acid) for 3–4 min and then in 60% fixative solution for 3 min on ice. The cells were collected by filtration using gauze and were then fixed with 1:1 ethanol/acetic acid. The cells in suspension were dropped onto glass slides and air-dried. The slides were then stained with 3% Giemsa for 10 min in phosphate buffer (pH 6.8).

For fluorescence microscopy to determine SC and the γ-H2AFX distribution, meiotic chromosomal slides were prepared using a drying-down method^63^. Briefly, seminiferous tubules were placed in a hypotonic extraction buffer containing 30 mM Tris-HCl, 50 mM sucrose, 17 mM trisodium citrate dehydrate, and 5 mM EDTA for 30 min on ice. The seminiferous tubules were minced in 100 mM sucrose, and the suspension was then dropped on 1% paraformaldehyde-dipped, MAS-coated glass slides (MATSUNAMI, S9116). The slides were placed in a humidified chamber to dry slowly. After 3 h, the slides were washed twice in 0.4% DRIWEL (Fujifilm) for 2 min and dried.

Immunostaining of the slides was performed as previously described, with some modifications^27^,^61^. The slides were immersed in 10% adb (antibody dilution buffer; 3% BSA, 0.05% Triton X-100 in PBS, pH 7.4) in PBS for 10 min, 10% adb/PBS with 0.05% Triton X-100 for 10 min, and 10% adb/PBS for 10 min. Primary antibodies were applied and incubated overnight at 4°C in a chamber with high humidity. After washing the slides using 10% adb/PBS with 0.2% DRIWEL, 10% adb/PBS, 10% adb/PBS with 0.05% Triton X-100, and 10% adb/PBS on ice for 10 min each, secondary antibodies were applied on the slides and incubated for 3 h at 4°C in a dark chamber with high humidity. The slides were washed with 0.2% DRIWEL in PBS and 0.05% Triton X-100 in PBS for 10 min each and twice with 0.2% DRIWEL for 5 min on ice with light interception. The slides were then mounted with 30 μl mounting solution containing 10 μl 1,4-diazabicyclo[2.2.2]octane, 10 μl *p*-phenylediamine, and 10 μl 4′,6-diamidino-2-phenylindole. Images of cells for each male were captured for quantitative analysis using a fluorescence microscope (Leica, as described below). Cells that appeared to reach the pachytene (parental species) and pachytene-like (hybrids) stages were randomly observed.

### Imaging

For immunocytochemistry, we used a cooled charge-coupled device (CCD) camera (Leica DFC360 FX, Leica Microsystems) mounted on a Leica DMRA microscope and analysed the data using the 550CW-QFISH application program of Leica Microsystems Imaging Solution. For light microscopy, we used an Olympus BX51 Microscope & DP70 Digital Camera System.

### Classification of stages in prophase I

We classified late zygotene and pachytene stages in the parental species based on the configuration and condensation of axial elements (AEs) and lateral elements (LEs). In the present study, we classified the late zygotene stage as the point at which nearly all of the AEs had synapsed extensively with their homologs and the AEs/LEs were condensed but longer than in the pachytene. In the pachytene, all of the AEs had completed chromosome synapsis, and the AEs/LEs were well condensed and shorter than in the zygotene. In the interspecific hybrids, cells similar to the late zygotene cells of parental species and cells in which the AEs and LEs were well condensed were classified as late zygotene- and pachytene-like cells, respectively. It should be noted that it is difficult to discriminate completely between late zygotene- and pachytene-like stages in the hybrids using the present method.

### Criteria for the determination of the synapsis and asynapsis of X and Y chromosomes

The synapsis and asynapsis of X and Y chromosomes in the pachytene spermatocytes of the parental species and pachytene-like spermatocytes of the F_1_ hybrids was examined by immunofluorescence analysis of LE configurations. When the LEs of the X and Y chromosomes had synapsed with each other, whether at their termini or extensively along the entire length of the Y chromosome, we regarded the chromosomal configuration as synapsis. When the LEs of the X and Y chromosomes were clearly separated, we regarded the chromosomal configuration as asynapsis. When we could not determine whether the sex chromosome axes were synapsed or separated, those pairs were judged as indeterminate. Notably, we were unable to distinguish using this method whether synapsis involved stable homologous pairing at PARs or nonhomologous association at chromosomal locations other than PARs in this method.

### Morphological analysis of spermatozoa

Spermatozoa were obtained from caudal epididymides and dispersed in 1 × PBS at 37°C. The sperm solution was diluted with 1 × PBS and stained with 1% Eosin Y for 20 min. After staining, two droplets of the sperm suspension were smeared on glass slides and air-dried. The slides were then observed under a light microscope.

### Statistical analysis

For the comparisons of testis weight, relative testis weight, and frequencies of spermatocyte degeneration at MI and dissociation of X and Y chromosomes at MI between parental species and their F_1_ hybrids, Welch's *t*-tests (two-sided) were performed. These tests were also used for the comparisons of the frequencies of asynapsis of the X and Y chromosomes in the pachytene spermatocytes immunostained with SYCP1 and SYCP3 antibodies and those immunostained with γ-H2AFX and SYCP3 antibodies. Welch's *t*-tests were also used to compare the frequencies of abnormal synapsis in autosomes, which included interlocking and partial synapsis between autosomal pairs and synapsis between autosomes and X or Y chromosomes, and abnormal synapsis in X and Y chromosomes. This included X-Y asynapsis and synapsis between autosomes and X or Y chromosomes in pachytene-like hybrid spermatocytes. We note that synapsis between autosomes and X or Y chromosomes was classified into abnormal synapsis both in autosomes and in X and Y chromosomes. To compare the frequencies of X-Y asynapsis and autosomal γ-H2AFX staining in pachytene-like hybrid spermatocytes, Welch's *t*-tests (two-sided) were also performed. A two-sided *P* value of less than 0.05 was considered significant in all tests.

## Author Contributions

S.I. and Kz.T. analysed all of the data in this study; Kz.T. performed all of the experiments, except for those parts of the histological analyses conducted by N.O.; S.I., Kz.T. and Y.M. wrote the manuscript, with comments from Km.T.; and Y.M. conceived and designed the study. S.I. and Kz.T. contributed equally.

## Supplementary Material

Supplementary Information Supplementary Information

## Figures and Tables

**Table 1:** Frequencies of X-Y chromosome dissociation in MI spermatocytes of *Phodopus campbelli, P. sungorus,* and their F_1_ hybrids

Animal	Number of individuals	Number of observed cells^a^	Frequency of cells with X-Y dissociation^b^ (%)
*P. campbelli*	2	100	7.0 ± 1.4
*P. sungorus*	2	100	6.0 ± 2.8
F_1_ (Type B)	4	200	93.5 ± 1.0
F_1_ (Type C)	9	450	90.2 ± 3.9

^a^A total of 50 cells were observed for each individual.

^b^Values are given as mean ± standard deviation.

**Table 2:** Frequencies of degenerated MI spermatocytes in *Phodopus campbelli, P. sungorus,* and their F_1_ hybrids

Animal	Number of individuals	Number of observed cells^a^	Frequency of degenerated cells^b^ (%)
*P. campbelli*	2	200	5.5 ± 0.7
*P. sungorus*	2	200	6.5 ± 2.1
F_1_ (Type B)	4	400	63.5 ± 1.3
F_1_ (Type C)	9	900	57.4 ± 4.5

^a^A total of 100 cells were observed for each individual.

^b^Values are given as mean ± standard deviation.

**Table 3:** Frequencies of asynapsis of X and Y chromosomes, association of X or Y chromosomes and autosomes, and association of autosome pairs in the pachytene spermatocytes of *Phodopus campbelli* and *P. sungorus* and pachytene-like spermatocytes of F_1_ hybrids

Animal	Number of observed cells	X-Y^a^	X//Y^b^	n.d.^c^	X or Y- Autosomes^d^	Autosomes-Autosomes^e^
*P. campbelli* (*n* = 2)	106	105	0	1	0	0
		99.09 ± 1.29%	0%	0.91 ± 1.29%	0%	0%
*P. sungorus* (*n* = 2)	147	147	0	0	0	0
		100%	0%	0%	0%	0%
F_1_ (Type B) (*n* = 6)	363	262	92	9	7	3
		70.88 ± 7.87%	26.92 ± 8.09%	2.20 ± 2.01%	2.30 ± 3.52%	0.75 ± 1.25%
F_1_ (Type C) (*n* = 5)	310	206	67	37	4	1
		67.57 ± 8.28%	20.26 ± 8.74%	12.17 ± 8.75%	2.16 ± 4.83%	0.54 ± 1.21%

^a^Number of cells with synaptic X and Y chromosomes.

^b^Number of cells with asynaptic X and Y chromosomes.

^c^Number of cells in which X and Y chromosome synapsis could not be determined.

^d^Number of cells that showed association between X or Y chromosomes and autosomes.

^e^Number of cells that showed association between autosome pairs.

*n*, number of animals; frequencies are given as mean ± standard deviation.

**Table 4:** Morphologies of XY bodies and frequencies of γ-H2AFX staining in autosomes and association between XY bodies and autosomes in the pachytene spermatocytes of *P. campbelli* and *P. sungorus* and pachytene-like spermatocytes of F_1_ hybrids

Animal	Number of observed cells	X-Y^a^	X//Y			γ-H2AFX in autosomes^e^	XY body-autosomes^f^
			Normal^b^	Broad^c^	Separated^d^		
*P. campbelli* (*n* = 2)	172	171	1	0	0	0	0
		99.40 ± 3.54%	0.60 ± 0.71%	0%	0%	0%	0%
*P. sungorus* (*n* = 2)	174	174	0	0	0	0	1
		100%	0%	0%	0%	0%	0.48 ± 0.68%
F_1_ (Type B) (*n* = 3)	199	136	48	7	8	12	5
		69.50 ± 17.31%	23.91 ± 12.56%	2.89 ± 3.58%	3.70 ± 3.21%	5.45 ± 3.71%	2.34 ± 2.03%
F_1_ (Type C) (*n* = 3)	221	175	42	2	2	7	4
		79.20 ± 9.06%	19.00 ±8.52%	1.03 ± 0.94%	0.78 ± 1.34%	3.24 ± 0.80%	2.01 ± 1.87%

^a^Number of cells with normal XY bodies and paired X and Y chromosomes.

^b^Number of cells with normal XY bodies and unpaired X and Y chromosomes.

^c^Number of cells with broad XY bodies comprising unpaired X and Y chromosomes.

^d^Number of cells with separated XY bodies and unpaired X and Y chromosomes.

^e^Number of cells containing autosomal regions stained with γ-H2AFX antibody. Staining signals on autosomes associated with the XY bodies were excluded.

^f^Number of cells that showed association between the XY bodies and autosomes.

*n*, number of animals; frequencies are given as mean ± standard deviation.
