# Supplementary material for: Abnormal pairing of X and Y sex chromosomes during meiosis I in interspecific hybrids of Phodopus campbelli and P. sungorus
Source: Sci Rep. 2015 Mar 24;5:9435. doi: 10.1038/srep09435 (PMC4371188; doi:10.1038/srep09435)
Supplement: Supplementary Information [file srep09435-s1.pdf]

Supplementary Information

Abnormal pairing of X and Y sex chromosomes during meiosis I in interspecific hybrids of *Phodopus campbelli* and *P. sungorus*

Satoshi Ishishita[1†], Kazuma Tsuboi[1†], Namiko Ohishi[2], Kimiyuki Tsuchiya[3], Yoichi Matsuda[1*]

[1] Laboratory of Animal Genetics, Department of Applied Molecular Biosciences, Graduate School of Bioagricultural Sciences, Nagoya University, Furo-cho, Chikusa-ku, Nagoya, Aichi 464-8601, Japan, [2] Laboratory of Animal Cytogenetics, Graduate School of Science, Hokkaido University, North 10 West 8, Kita-ku, Sapporo, Hokkaido 060-0810, Japan, [3] OOYO SEIBUTSU Inc., Minato-ku, Tokyo 107-0062, Japan

[†] These authors contributed equally to this work

* Corresponding author.

Table S1. Body weight, testis weight, and relative testis weight in *Phodopus campbell, P. sungorus,* and their F_1 hybrids

Animal	Number of individuals	Body weight[a] (g)	Testis weight[a] (mg)	Relative testis weight[a,b]
P. campbelli	3	33.6 ± 5.8	1109.3 ± 145.3	3.33 ± 0.38
P. sungorus	3	32.9 ± 5.6	957.0 ± 67.5	2.95 ± 0.43
F_1	29	19.8 ± 2.2	176.8 ± 143.5	0.89 ± 0.72
F_1 (Type A)	9	19.8 ± 3.0	27.2 ± 8.7	0.14 ± 0.05
F_1 (Type B)	9	19.5 ± 2.1	142.6 ± 77.9	0.72 ± 0.35
F_1 (Type C)	11	20.1 ± 1.7	327.2 ± 81.9	1.65 ± 0.41

[a] Values are given as mean ± standard deviation.

[b] Testis weight (g)/body weight (g) × 100.

Table S2. Quantification of asynapsis of X and Y chromosomes, association of X or Y chromosomes and autosomes, and association of autosome pairs in the pachytene spermatocytes of *Phodopus campbelli* and *P. sungorus* and pachytene-like spermatocytes of F_1 hybrids

Animal	ID	Number of observed cells	X-Y[a]	X//Y[b]	n.d.[c]	XY-Autosomes[d]	Autosomes-Autosomes[e]
P. campbelli	110315	55	54	0	1	0	0
			98.18%	0%	1.82%	0%	0%
	110324	51	51	0	0	0	0
			100%	0%	0%	0%	0%
	total	106	105	0	1	0	0
			99.09 ± 1.29%	0%	0.91 ± 1.29%	0%	0%
P. sungorus	110307	45	45	0	0	0	0
			100%	0%	0%	0%	0%
	110310	102	102	0	0	0	0
			100%	0%	0%	0%	0%
	total	147	147	0	0	0	0
			100%	0%	0%	0%	0%
F1 (Type B)	1-1	44	30	14	0	4	0
			68.18%	31.82%	0%	9.09%	0%
	2-2n	56	35	20	1	0	0
			62.50%	35.71%	1.79%	0%	0%
	3-1n	66	53	13	0	0	1
			80.30%	19.70%	0%	0%	1.52%
	6-1	90	73	13	4	2	0
			81.11%	14.44%	4.44%	2.22%	0%
	14-1n	40	27	12	1	1	0
			67.50%	30.00%	2.50%	2.50%	0%
	20-3	67	44	20	3	0	2
			65.67%	29.85%	4.48%	0%	2.99%
	total	363	262	92	9	7	3
			70.88 ± 7.87%	26.92 ± 8.09%	2.20 ± 2.01%	2.30 ± 3.52%	0.75 ± 1.25%
F_1 (Type C)	3-2	92	63	28	1	0	0
			68.48%	30.43%	1.09%	0%	0%
	3-3n	44	35	3	6	0	0
			79.55%	6.82%	13.64%	0%	0%
	8-1	76	46	14	16	0	0
			60.53%	18.42%	21.05%	0%	0%
	6-2	61	36	13	12	0	0
			59.02%	21.31%	19.67%	0%	0%
	18-1n	37	26	9	2	4	1
			70.27%	24.32%	5.41%	10.81%	2.70%
	total	310	206	67	37	4	1
			67.57 ± 8.28%	20.26 ± 8.74%	12.17 ± 8.75%	2.16 ± 4.83%	0.54 ± 1.21%

[a] Number of cells with synaptic X and Y chromosomes.
[b] Number of cells with asynaptic X and Y chromosomes.
[c] Number of cells in which X and Y chromosomes synapsis could not be determined.
[d] Number of cells that showed association between X or Y chromosomes and autosomes.
[e] Number of cells that showed association between autosome pairs.
Frequencies are given as mean ± standard deviation.

Table S3. Morphologies of XY bodies and quantification of γ-H2AFX staining in autosomes and association between the XY bodies and autosomes in the pachytene spermatocytes of *Phodopus campbelli* and *P. sungorus* and pachytene-like spermatocytes of F_1 hybrids

Animal	ID	Number of observed cells	X-Y[a]	X//Y			γ-H2AFX in autosomes[e]	XY-autosomes association[f]
				(Normal)[b]	(Broad)[c]	(Separated)[d]		
P. campbelli	110324	84	83	1	0	0	0	0
			98.81%	1.19%	0%	0%	0%	0%
	110315	88	88	0	0	0	0	0
			100%	0%	0%	0%	0%	0%
	total	172	171	1	0	0	0	0
			99.40 ± 3.54%	0.60 ± 0.71%	0%	0%	0%	0%
P. sungorus	110307	70	70	0	0	0	0	0
			100%	0%	0%	0%	0%	0%
	110310	104	104	0	0	0	0	1
			100%	0%	0%	0%	0%	0.96%
	total	174	174	0	0	0	0	1
			100%	0%	0%	0%	0%	0.48 ± 0.68%
F_1 (Type B)	4-a-1	56	32	20	1	3	3	2
			57.14%	35.71%	1.79%	5.36%	5.36%	3.57%
	6-1	87	54	22	6	5	8	3
			62.07%	25.29%	6.90%	5.75%	9.20%	3.45%
	20-3	56	50	6	0	0	1	0
			89.29%	10.71%	0%	0%	1.79%	0%
	total	199	136	48	7	8	12	5
			69.50 ± 17.31%	23.91 ± 12.56%	2.89 ± 3.58%	3.70 ± 3.21%	5.45 ± 3.71%	2.34 ± 2.03%
F_1 (Type C)	1-a-2	86	61	23	0	2	2	2
			70.93%	26.74%	0%	2.33%	2.33%	2.33%
	18-1	54	42	11	1	0	2	2
			77.78%	20.37%	1.85%	0%	3.70%	3.70%
	a-1	81	72	8	1	0	3	0
			88.89%	9.88%	1.23%	0%	3.70%	0%
	total	221	175	42	2	2	7	4
			79.20 ± 9.06%	19.00 ± 8.52%	1.03 ± 0.94%	0.78 ± 1.34%	3.24 ± 0.80%	2.01 ± 1.87%

[a] Number of cells with normal XY bodies and synapsed X and Y chromosomes.

[b] Number of cells with normal XY bodies and unsynapsed X and Y chromosomes.

[c] Number of cells with broad XY bodies comprising unsynapsed X and Y chromosomes.

[d] Number of cells with separated XY bodies and unsynapsed X and Y chromosomes.

[e] Number of cells containing autosomal regions stained with γ-H2AFX antibody. Staining signals on autosomes associated with the XY body were excluded.

[f] Number of cells that showed association between the XY bodies and autosomes.

n , number of animals; frequencies are given as mean ± standard deviation.

Table S4. Morphological abnormalities of spermatozoa in *Phodopus campbelli, P. sungorus,* and their F_1 hybrids.

Morphology of spermatozoa	*P. campbelli*	*P. sungorus*	F_1 (Type C)
Normal	176	180	14
Shelving curve hook	2	2	176
Bent hook	12	5	135
Thick hook	9	11	93
Fallen head with short hook	0	2	38
Short hook	0	0	10
Other	1	0	34
Total (%)[a]	200 (12.0)	200 (10.0)	500 (97.2)

[a] Frequencies of abnormal sperm heads.

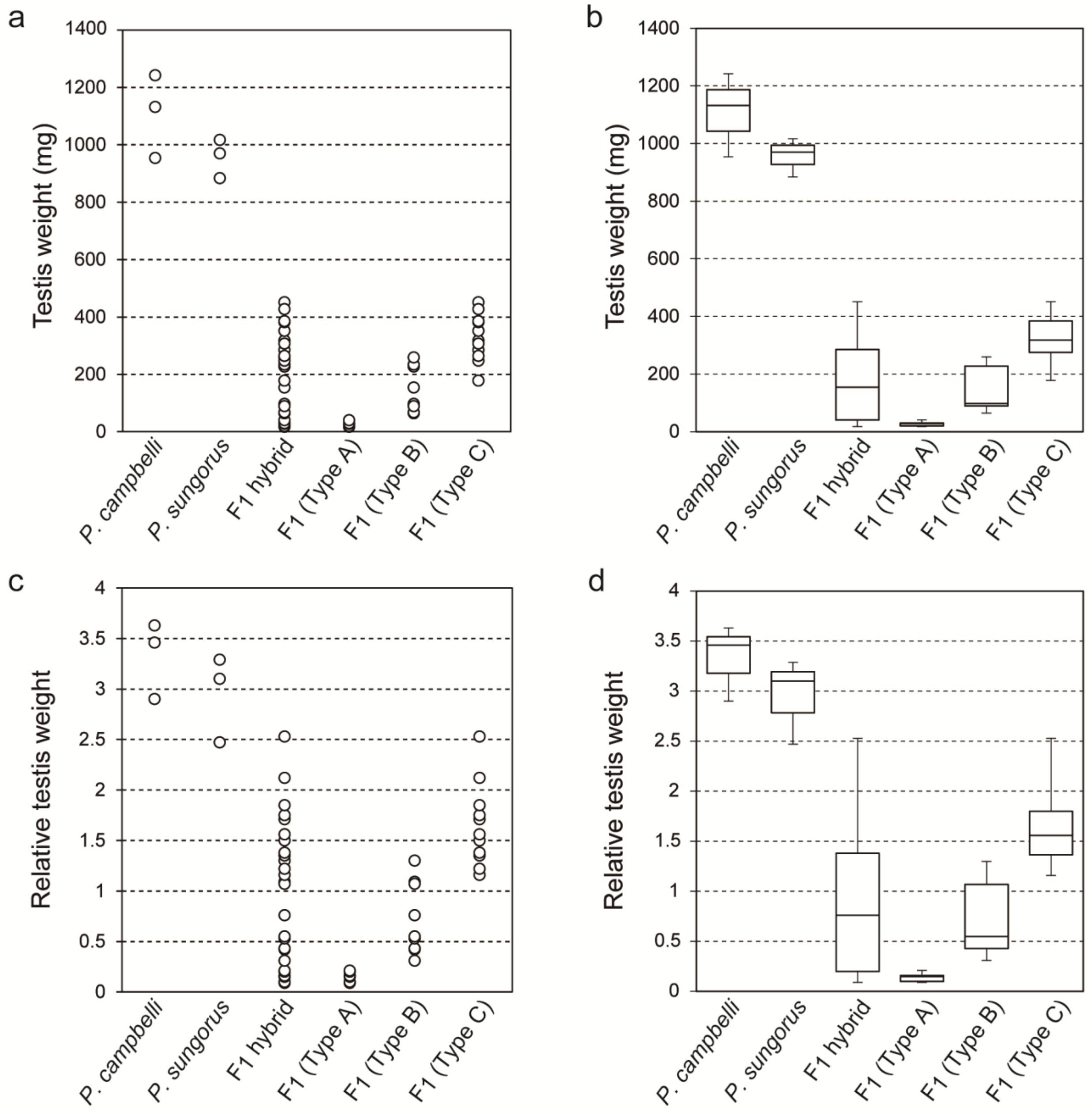

Figure S1. Testis weight and relative testis weight of *Phodopus campbelli, P. sungorus*, and their F_1 hybrids. a–d Testis weight and relative testis weight of *P. campbelli, P. sungorus,* and Type A, Type B, and Type C hybrids are shown by the dot blots (**a, c**) and box-and-whisker plots (**b, d**). The bottom and top of the box represent the first and third quartiles, the line inside the box represents the median, and the ends of the whiskers represent the minimum and maximum values.
